# Editorial: Methods and applications in educational psychology

**DOI:** 10.3389/fpsyg.2023.1225744

**Published:** 2023-06-13

**Authors:** María Soledad Segretin, Mei-Shiu Chiu, Kai-Yu Tang, Antonio P. Gutierrez de Blume, Christelle Declercq

**Affiliations:** ^1^Unit of Applied Neurobiology (UNA, CEMIC-CONICET), National Scientific and Technical Research Concul (CONICET), Buenos Aires, Argentina; ^2^College of Education, National Chengchi University, Taipei City, Taiwan; ^3^Graduate Institute of Library & Information Science, National Chung Hsing University, Taichung, Taiwan; ^4^Department of Curriculum, Foundations, and Reading, Georgia Southern University, Statesboro, GA, United States; ^5^Université de Reims Champagne-Ardenne, France, Laboratoire C2S (Cognition, Santé, Société) EA6291, Reims, France

**Keywords:** educational assessment and evaluation, natural settings, mixed-methods, learning strategies, individual differences, academic performance and behavior, educational resources

This Research Topic aims to highlight the latest research methods used to investigate fundamental questions in Educational Psychology. The 14 contributions included in this Research Topic bring major conceptual and methodological advances in six Research Topics, in terms of methodology use.

A challenge for this field of research, and the first theme for this Research Topic of articles, addressed methodological issues. These studies use diverse novel methodologies. Magliano et al. examined how theoretically motivated and computationally derived indices of cohesion can be used to explore aspects of coherence-building and how these measures relate to college students' foundational reading skills. Based on the analysis of a large sample, results suggested that the computational analysis of constructed response associated with coherence-building strategies is correlated with individual differences in foundational reading skills. In addition, the relations between foundational reading skill and response cohesion were relatively stable across contexts (i.e., text genre and institutions). Borgstede and Rau propose a new approach to the problem of integration in mixed methods research that builds on a representational understanding of empirical science. From this perspective, qualitative and quantitative modeling strategies constitute two different ways to represent empirical structures. Whereas, qualitative representations focus on the construction of types from cases, quantitative representations focus on the construction of dimensions from variables. Authors argue that types and dimensions should be integrated within a joint representation of the data that equally acknowledges qualitative and quantitative aspects. Fu et al. introduce the R package SSDbain, which can be used to calculate the sample size required to evaluate hypotheses using the Approximate Adjusted Fractional Bayes Factor (AAFBF) for one-way ANOVA models, as implemented in the R package bain. The Bayesian ANOVA, Bayesian Welch's ANOVA, and Bayesian robust ANOVA are available. Using the R package SSDbain and/or the tables provided in this paper, researchers in the social and behavioral sciences can easily plan the sample size if they intend to use a Bayesian ANOVA. Including a neural level of analysis, Ayabe et al. study the development of diagram use competence following the provision of task-appropriate instruction. They focused on both behavioral and neurophysiological evidence (i.e., brain activity, using functional near-infrared spectroscopy or fNIRS). The study was implemented in a sample of children and adolescents (10–19 years of age) and participants were asked to solve mathematical word problems for which the use of tables (which is one kind of diagram) was deemed effective. Results demonstrate important neurophysiological changes resulting from task-appropriate instruction that promotes effective strategy use and improves learning performance.

The second group of articles addressed the role of different individual characteristics in student academic life and learning, using survey and related quantitative data analysis methods. In particular, Shi and Qu analyzed the moderating role of self-monitoring in the association between cognitive abilities (i.e., memory, representational, information processing, logical reasoning, and thinking conversion), and academic achievement in a sample of adolescent students. The results showed that cognitive ability can positively affect academic achievement, while self-monitoring moderate the effect of cognitive ability on academic performance, in particular on mathematics and English subjects. By his part, Zhang et al. implement a survey in a large sample of college students to examine the mediating role of self-concept and social support in the associations between physical exercise and depression. The results suggest that physical exercise negatively predicted college depression. Moreover, self-concept and social support mediate the relationship between physical exercise and depression.

The third group of articles aimed to propose adaptation, validation and/or development of instruments for specific educational assessment. Yang et al. describe the initial development and validation of the Student Conceptual Level Scale with a large sample of students from secondary schools. The authors constructed a three-factor model (learning awareness level, autonomous input level, and environmental coping level), each with its own independent set of items. This study validated the use of full-scale and subscale scores and examined their relationship with different validity criteria (i.e., autonomous learning, mental effort, and academic scores). This updated measure reflects the value and role of the conceptual level in the learning and individual development of students, and provides a more complete frame of reference for the use of the conceptual level in teaching and learning. Gonzales-Valdivia et al. adapted to Spanish and evaluate the psychometric properties of the H-Sat Scale to assess satisfaction with school in Peruvian students between 11 and 18 years old. The scale presented adequate internal consistency for each of the five factors (confirmed through a confirmatory analysis). This measurement tool could be used for the evaluation of interventions in school and health contexts to assess other aspects of wellbeing necessary for their development in school-age students.

The fourth group of articles addressed conceptual or theoretical models or frameworks in the field. Jirout et al. highlighted the importance of curiosity in children's development and academic performance, which has been widely acknowledged but not extensively studied in classroom settings. The result of the feasibility test showed that the Curiosity in Classrooms (CiC) framework is a useful tool that can help teachers to support students' development and academic success. The authors begin by describing the framework they used to identify specific instructional practices that can promote curiosity, and then focus on the development of a coding protocol for and test of the framework. Sun proposed the construction of an educational resource recommendation model based on Takagi and Sugeno (T–S) fuzzy neural network, verified the feasibility of the model, combined the educational resource recommendation model with university teaching, and analyzed the application effect. After applying the educational resource recommendation model, the accuracy of educational resource recommendation is improved, and the design is feasible. The educational management mode with positive psychological emotions has a good teaching effect, which can improve teachers' dedication and concentration. Teaching resource recommendation mode can improve college students' interest in the application of teaching resources to a certain extent, and their application satisfaction is improved. Based on learning strategy models and using thematic analysis, Liu and Uesaka proposed a new lecture note-taking framework comprising shallow and deep lecture note-taking. For that, data from high school students from two countries (Japan and China) were analyzed to explore cognitive activities in which students engage when taking lecture notes in mathematics classes. Results provided insights into the cognitive activities accompanying lecture note-taking, such as the metacognitive function, which has yet to be explored in previous research. In addition, the authors inferred that the differences in lecture notes might result from the influence of the student's beliefs and teachers' instruction styles.

The fifth, Annamalai et al. used a qualitative case study approach to understand the factors that affect students' motivation and satisfaction with online assessments in three different countries (Malaysia, Lithuania, and Spain) during the COVID-19 pandemic. The finding highlighted the importance of effective assessment guidelines and feedback in online learning environments. In addition, the impact of technology is also suggested for the investigation in the contexts of online assessment, such as virtual reality or gamification, and how they impact student motivation and engagement.

The sixth, two literature review methodologies are used to review papers in this Research Topic. Shaojie et al. discuss, based on a systematic literature review, the effects of audiovisual input on second language acquisition and the factors that influence the difficulty of audiovisual learning. The authors concluded that audiovisual input could provide more authentic language input and more adequate and richer multimodal cultural and situational contexts, which can better promote learners' understanding of the content and stimulate learners' interest in participating in listening comprehension tasks. Factors affecting audiovisual multimodal input difficulty included subtitles, video inputs, and sounds and pictures relationships. Lin et al. review the research trends of scaffolding in the field of science education. Then, descriptive and co-word analysis were conducted to examine the selected articles published in the Social Science Citation Index journals in the past 20 years. Overall, this study reveals a growing trend of science educators' academic publications about scaffolding in the recent two decades. In addition, results showed that “scaffolding,” “support,” and “design” were the top three most frequently used keywords during 2000 and 2019. Visualization of co-word networks in each 5-year period further helps clarify both educators' common research focuses and relevant research trends.

In summary, the 14 papers included in this Research Topic focus on six major Research Topics and using diverse methodologies. The first group has four papers, which directly deal with the issue of methodologies in the field, including natural language processing, mixed-methods integration, sample size determination, and behavioral -neurophysiological data integration. The second group (with two papers) focuses on students' academic life, using survey as the major methodology. The two papers in the third group focuses on scale development. The three papers in the fourth group focuses on framework building and further exploration or examination. One paper uses a qualitative, interview method, and two papers using two different literature review methods. This Research Topic collection may mimic the present research fronts and suggest developing more diverse, novel methodologies to study topics in relation to learning, instruction, and assessment in educational psychology. Multi-, inter-, and transdisciplinary endeavor may be needed to advance a deeper understanding of the field and to face human challenges in the world (e.g., artificial intelligence and the pandemic).

## Author contributions

MSS and M-SC: writing original draft. K-YT, APGB, and CD: writing review. All authors contributed to the article and approved the submitted version.

